# Clinical Detection and Management of Colorectal Cancer Concerning the Gut Microbiome

**DOI:** 10.1155/sci5/1945321

**Published:** 2025-09-08

**Authors:** Hamed Eraghieh Farahani, Maryam Pourhajibagher, Abbas Bahador

**Affiliations:** ^1^Department of Microbiology, School of Medicine, Tehran University of Medical Sciences, Tehran, Iran; ^2^Dental Research Center, Dentistry Research Institute, Tehran University of Medical Sciences, Tehran, Iran

**Keywords:** chemotherapy, colorectal cancer (CRC), gut microbiome, immunotherapy, probiotics

## Abstract

Colorectal cancer (CRC) is a prevalent malignancy worldwide and a leading cause of cancer-related mortality, influenced by both genetic predisposition and environmental factors. Gut dysbiosis, characterized by an imbalance in the gut microbiome, has been identified as a significant contributor to CRC progression. Although considerable progress has been made in understanding the relationship between the gut microbiome and CRC, the precise underlying mechanisms remain incompletely elucidated. Recent studies emphasize the role of gut microorganisms in inducing DNA damage, promoting inflammation, and contributing to drug resistance, positioning the microbiome as a promising target for CRC prevention and therapy. This review examines the intricate relationship between gut microbiota and CRC, with a focus on tumorigenesis mechanisms and the potential utility of specific bacterial species as clinical biomarkers. Dysbiosis, often driven by dietary and environmental factors, has been implicated in CRC pathogenesis, with bacterial virulence factors, inflammatory pathways, and microbial metabolites playing central roles in disease progression. Strategies for modulating the gut microbiome, such as probiotic supplementation and other microbiome-targeted interventions, represent emerging therapeutic approaches. Additionally, this review discusses the challenges associated with translating microbiome research into clinical practice and proposes potential solutions. By advancing the understanding of microbiota-CRC interactions, this research offers valuable insights into novel strategies for CRC prevention, early detection, and treatment. Future studies aim to refine microbiome-based interventions, ultimately improving the clinical management of CRC.

## 1. Introduction

Colorectal cancer (CRC) is a major global health issue, responsible for 10% of cancer cases and approximately 700,000 deaths annually [1]. In Slovenia, it is among the most prevalent malignancies [2]. Nearly 90% of CRC cases are sporadic, though risk factors include genetics and lifestyle. Lifestyle risks encompass obesity, smoking, alcohol consumption, physical inactivity, and diets high in fat and processed meats but low in fiber [3–5]. Genetic predispositions, such as adenomatous polyposis, Lynch syndrome, and Peutz–Jeghers syndrome, also increase CRC risk by promoting the progression of polyps into tumors. Environmental factors are significant in CRC, with heritability estimates of 12%–35%, underscoring the role of nongenetic influences, particularly gut microbiota, in CRC development [6–8]. In 2012, over 15% of cancer cases were linked to infectious pathogens [9]. The gut microbiota interacts with the cells of the colon, influencing immunity, metabolism, and inflammation through metabolites such as short-chain fatty acids (SCFAs) and polyamines. Dysbiosis, or microbial imbalance, disrupts these functions, contributing to the progression of CRC [9–11].

Pathogenic bacteria such as *Escherichia coli*, *Bacteroides fragilis*, and *Fusobacterium nucleatum* exacerbate CRC by inducing chronic inflammation and promoting tumor growth. *F. nucleatum*, for example, has been associated with resistance to oxaliplatin treatment [12–16]. Advances in microbiota research have improved CRC detection by integrating stool microbiome analysis with traditional screening methods like fecal occult blood tests (gFOBTs) and fecal immunochemical tests (FITs) [17, 18]. However, translating microbiota-based therapies into clinical practice remains a significant challenge.

This review examines the role of the gut microbiota in CRC, with a focus on mechanisms revealed in experimental studies that have translational potential. While promising, further research is needed to harness the microbiome fully for the diagnosis and treatment of CRC.

## 2. The Gut Microbiota and CRC

### 2.1. Diversity of the Gut, The Microbial Community in CRC

Experimental studies on animals have highlighted the significant role of gut microbiota in CRC. Research dating back to the 1960s, which compared germ-free (GF) and conventional mice exposed to carcinogens, demonstrated that gut bacteria are essential for the carcinogenic effects of cycasin, as cancer did not develop in GF mice under identical conditions. Subsequent investigations identified β-glucosidase enzymes in the gut as key mediators in the hydrolysis of cycasin into methylazoxymethanol (MAM), the active carcinogenic compound. Furthermore, conventional rats exhibited a higher incidence of MAM-induced intestinal tumors compared to GF rats, reinforcing the critical contribution of gut microbiota to CRC pathogenesis. [19]. Recent studies have enhanced our understanding of the mechanisms by which specific bacterial species contribute to CRC progression. One investigation examined the impact of *F. nucleatum* on epithelial-mesenchymal transition (EMT) using the human normal colon epithelial cell line NCM460. The results demonstrated that *F. nucleatum* promotes cell proliferation and invasion while markedly increasing NF-κB activation through phosphorylation of its p65 subunit. This activation subsequently leads to elevated levels of proinflammatory mediators, including interleukin (IL)-6, IL-1β, and matrix metalloproteinase (MMP)-13. Notably, *F. nucleatum* did not affect β-catenin or E-cadherin expression levels but instead interacted with E-cadherin to enhance malignancy. Silencing E-cadherin impaired the bacterium's ability to induce inflammation and drive cell cycle progression, suggesting that *F. nucleatum* exploits E-cadherin to facilitate CRC development and may represent a promising target for antimicrobial therapeutic strategies [20].

Another study highlighted the enrichment of Fusobacterium species in human colonic adenomas and CRC tissues compared to healthy controls. Using an ApcMin/+ mouse model, researchers found that F. nucleatum not only increased tumor formation but also selectively recruited myeloid cells, creating a proinflammatory tumor microenvironment. This inflammatory profile closely resembled that observed in human Fusobacteria-positive CRC cases. Additionally, F. nucleatum promoted tumorigenesis even in the absence of colitis, with its associated gene expression signature correlating with increased NF-κB activation in human CRC [21]. The broader role of gut microbiota in CRC has been extensively reviewed. Dysbiosis—an imbalance in gut microbial composition—has been linked to CRC development, with certain bacteria, such as B. fragilis and F. nucleatum, being associated with an increased cancer risk. Gut bacteria can metabolize dietary procarcinogens into active carcinogens and activate oncogenic signaling pathways. Conversely, beneficial microbial metabolites, such as SCFAs, particularly butyrate, exhibit antitumor properties. Probiotics have been proposed as potential CRC preventive agents due to their ability to modulate gut microbiota composition favorably [22].

Further evidence supporting the role of the gut microbiome in CRC comes from a study utilizing a mouse model of inflammation-induced CRC, induced by azoxymethane (AOM) and dextran sodium sulfate (DSS). The study demonstrated significant alterations in gut microbial composition associated with tumorigenesis. Notably, antibiotic treatment (ABX) led to a reduction in both tumor size and number, highlighting the microbiome's involvement in cancer progression. Moreover, GF mice colonized with microbiota from tumor-bearing mice developed significantly more tumors compared to those receiving microbiota from healthy mice. Specific microbial shifts were linked to tumor development, including an increase in *Bacteroides*, *Odoribacter*, and *Akkermansia*, alongside a reduction in Prevotellaceae and Porphyromonadaceae. These findings provide direct evidence that gut microbiota alterations actively contribute to CRC progression, particularly in inflammation-driven contexts [23].

**Figure 1:**
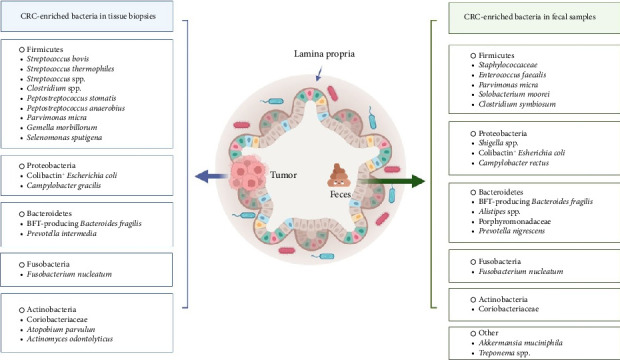
The presence of specific bacterial species in tissue biopsies and fecal samples from CRC patients suggests their potential as diagnostic biomarkers. For instance, combining fecal *Fusobacterium nucleatum* and *Clostridium symbiosum* detection with the fecal immunochemical test (FIT) significantly enhances diagnostic accuracy for advanced adenoma and CRC. Additionally, the sensitivity of FIT in detecting advanced adenoma improves when enriched bacterial genera linked to CRC, such as *Fusobacterium*, *Peptostreptococcus*, *Porphyromonas*, *Prevotella*, *Parvimonas*, *Bacteroides*, and *Gemella*, are identified. These findings highlight the importance of CRC-associated bacteria as diagnostic tools and their role in advancing early detection efforts. The figure is an original figure created by the authors of the manuscript.

Advances in metagenomics have enabled detailed microbiome profiling, revealing that microbial dysbiosis is a hallmark of CRC. Patients with CRC often exhibit increased levels of procarcinogenic species, such as *B. fragilis*, *Enterococcus faecalis*, *E. coli*, *F. nucleatum*, and *Peptostreptococcus anaerobius*, alongside reduced protective species, including *Clostridium butyricum* and *Bifidobacterium* [4, 24, 25]. This imbalance reduces the number of butyrate-producing bacteria and increases the presence of proinflammatory pathogens, disrupting gut homeostasis and promoting tumor growth [26, 27]. For instance, stool samples from CRC patients exhibit lower bacterial diversity, characterized by elevated levels of *F. nucleatum* and *Porphyromonas*, as well as fewer Gram-positive, fiber-fermenting *Clostridia* [28]. The gut microbiota also includes viral and fungal communities. Tumor tissues have higher viral DNA loads, implicating viruses such as human *papillomaviruses*, *polyomaviruses*, and *herpes* viruses in CRC. Next-generation sequencing (NGS) studies link CRC to increased virome diversity, including *Orthobunyavirus*, *Inovirus*, and *Tunalikevirus*, which target cancer-associated bacteria such as *B. fragilis* and *F. nucleatum* [29, 30]. Lysogenic bacteriophages, particularly those from the Siphoviridae and Myoviridae families, alter gut bacterial communities by inducing lysis, allowing cancer-associated microbes to form biofilms that drive inflammation and tumorigenesis [31]. The fungal microbiome also plays a role in CRC. Fungal species, such as *Phoma* and *Candida*, are more abundant in colorectal adenomas (CRA). In contrast, CRC patients exhibit a higher Basidiomycota-to-Ascomycota ratio and an increase in *Malasseziomycetes*. Conversely, *Saccharomyces cerevisiae*, known for its anti-inflammatory properties, is reduced in CRC patients, suggesting its therapeutic potential [32, 33] (Figure 1).

These studies revealed that CRC arises from an imbalance in gut bacterial composition, coupled with disruptions in the stability of the gut virome and mycobiome.

### 2.2. Intestinal Microbiome, Inflammation, Immunological Modulation, and Colon Cancer

The intestinal tract plays a central role in mediating the interaction between the immune system and gut microbiota, a relationship intricately linked to the development of CRC [34]. Chronic inflammation, estimated to contribute to approximately 20% of all cancers, is a key driver of CRC progression [35]. In CRC, inflammatory cytokines and chemokines foster a tumor-supportive microenvironment by recruiting myeloid progenitor cells and helper T cells, which in turn promote tumor growth through the release of growth factors, induction of angiogenesis, activation of tissue remodeling enzymes, and suppression of antitumor T-cell responses [36–39].

Gut dysbiosis and increased intestinal permeability are strongly associated with colon inflammation, a significant contributor to CRC pathogenesis [40, 41]. Increased permeability allows bacterial components like lipopolysaccharides (LPS) to trigger immune responses, initiating cytokine-driven inflammatory cascades that enhance tumor growth [39]. Specific gut microbes intensify these effects. *F. nucleatum* activates the NF-κB pathway and facilitates myeloid cell infiltration, while enterotoxigenic *B. fragilis* (ETBF) produces a toxin that activates IL-17 and NF-κB signaling, fostering a proinflammatory environment [21, 42]. Other bacteria such as pks-positive *E. coli*, *E. faecalis*, and *Alistipes finegoldii* also contribute to tumorigenesis via inflammation [43–46]. Gut microbes produce chemotactic factors, including CXCL9, CXCL10, CCL17, and CCL20, that recruit cytotoxic and IL-17-producing helper T cells (Th17) to tumor sites [47]. Inflammatory markers such as TNF-α, IL-1, CXCL2, and IL-17 are overexpressed in CRC, underscoring the link between inflammation and tumor progression [48].

Alterations in the gut microbiome, particularly involving pathogenic bacteria, are central to CRC development. Several species—including *Bacteroides vulgatus*, *Bacteroides thetaiotaomicron*, *Bacteroides longum*, *Eubacterium rectale*, *Ruminococcus gnavus*, *Faecalibacterium prausnitzii*, *B. fragilis*, and *Streptococcus gallolyticus*—have been identified in stool and tumor samples from CRC patients, making them potential biomarkers due to their association with tumorigenic processes [49–51]. These bacteria influence CRC through mechanisms such as immune modulation, epithelial proliferation, barrier dysfunction, inflammation, and metabolic changes. For example, *B. fragilis* toxin (BFT) activates Wnt and NF-κB signaling pathways, increasing inflammatory molecule production and tumor progression.

Dysbiosis, consistently reported in CRC patients, often features an overrepresentation of genera like *Fusobacterium*, *Peptostreptococcus*, *Porphyromonas*, *Prevotella*, *Parvimonas*, *Bacteroides*, and *Gemella* [24]. A case-control study analyzing fecal microbiota from 47 CRC patients and 94 healthy controls using 16S rRNA gene sequencing found reduced microbial diversity and a decreased abundance of *Clostridia*, alongside elevated levels of *Fusobacterium* and *Porphyromonas* in CRC cases. These results, confirmed via qPCR, suggest that gut microbiota composition may represent a modifiable risk factor in CRC prevention [28]. The microbiota's influence extends to immunity, cancer immunotherapy, and responses to immune checkpoint blockade. Dysbiosis can impair immunosurveillance and affect therapeutic outcomes, prompting interest in microbiome-based interventions such as dietary strategies, bacterial consortia, and fecal microbiota transplantation (FMT) [41].

Reviews have also highlighted the involvement of microbial biofilms, barrier dysfunction, and obesity in CRC, while emphasizing the therapeutic potential of microbiota modulation [52]. Mechanistic studies have provided deeper insights into bacterial genotoxins and their impact on CRC. BFT upregulates spermine oxidase (SMO) in colonic epithelial cells, leading to increased reactive oxygen species (ROS) and DNA damage—effects that were reduced by SMO inhibition in mouse models [53]. BFT binds to intestinal epithelial cells through a receptor-mediated, cholesterol-dependent mechanism, independent of E-cadherin or PARs, initiating downstream signaling [54]. *E. coli* carrying the pks island produces colibactin, a genotoxin that crosslinks DNA, promoting inflammation and mutations [55]. Other genotoxins, like cytolethal distending toxin (CDT) from *E. coli* and *Campylobacter* spp., and AvrA protein from *Salmonella*, cause DNA damage and activate carcinogenic signaling pathways [55–58]. *F. nucleatum* specifically targets tumor tissues via its surface protein Fap2, which binds Gal-GalNAc on CRC cells, and its adhesin FadA, which activates β-catenin signaling through E-cadherin, inducing inflammation and carcinogenesis [59, 60]. Together with pks-positive *E. coli*, these bacteria contribute significantly to CRC by fostering an inflammatory environment and promoting epithelial transformation [57, 61]. This growing body of evidence underscores the microbiota's role in modulating immune responses and promoting conditions conducive to CRC, positioning it as both a biomarker and a potential therapeutic target. However, while mechanistic insights support a role for the microbiome in carcinogenesis, direct evidence for causation remains limited. A consensus panel emphasized that the microbiome is one component within a complex interplay of environmental and host factors, and large-scale longitudinal studies are needed to elucidate its definitive role in cancer development [62].

**Figure 2:**
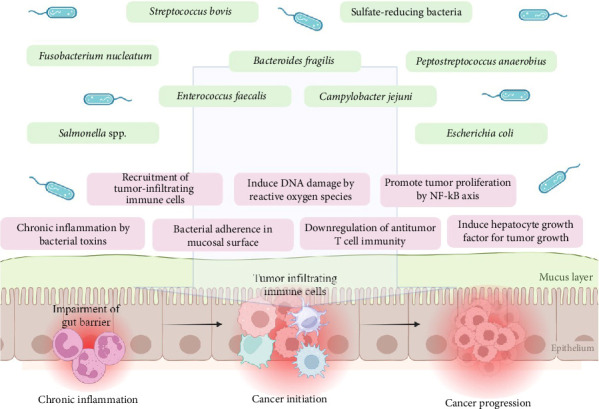
The development of CRC is driven by a complex interaction between the intestinal microbiome and tumor progression. Certain gut microbes contribute to CRC by triggering chronic inflammation in the colorectal epithelium. For example, *Salmonella* produces typhoid toxin, and *Escherichia coli* generates colibactin, both of which stimulate proinflammatory cytokine production and enhance bacterial adherence to epithelial cells. This sustained inflammation creates a tumor-supportive microenvironment, a key factor in CRC pathogenesis. Microbial activity also leads to increased reactive oxygen species (ROS) and DNA damage in epithelial cells, further promoting carcinogenesis. Additionally, bacteria such as *Fusobacterium nucleatum* and *Bacteroides fragilis* influence the immune microenvironment to favor tumor progression. These microbes reduce CD3+ T-cell density, weakening antitumor immune responses while encouraging the recruitment and proliferation of proinflammatory T-helper cells. This immune modulation fosters conditions that support tumor growth, emphasizing the critical role of the gut microbiome in CRC development.

These findings highlight the pivotal role of specific bacteria and their virulence factors in transforming healthy colonic epithelial cells into cancerous cells (Figure 2).

## 3. CRC, Metabolites, and Gut Microbiome

The intestinal microbiome produces various metabolites through the anaerobic fermentation of undigested food, influencing immune responses, epithelial interactions, and disease development. Among these are potentially carcinogenic compounds like polyamines from protein fermentation [63, 64]. SCFAs—primarily acetate, propionate, and butyrate—are key metabolites produced by gut microbiota through fermentation of dietary fibers and resistant starches [65, 66]. Butyrate supports gut health by activating the GPR109a receptor in epithelial cells, which induces IL-18 synthesis, aiding mucosal healing and maintaining gut barrier integrity [67]. However, CRC patients often exhibit reduced levels of butyrate-producing bacteria [63, 68]. This reduction is strongly associated with increased CRC risk and progression. Butyrate exhibits antitumor effects by inducing apoptosis, halting proliferation, and modulating gene expression through histone modification and DNA methylation. SCFAs also regulate inflammation by inhibiting NF-κB and activating G-protein-coupled receptors, including GPR43 and GPR109A. Additionally, they influence the Wnt/β-catenin pathway and contribute to systemic effects, such as appetite control and gut–brain signaling. Recent findings also show SCFAs enhance the release of extracellular vesicles (EVs) from CRC cells, enabling detection of microsatellite instability (MSI), a valuable biomarker for noninvasive cancer diagnostics. Altogether, these findings underscore the protective and diagnostic potential of SCFAs in CRC prevention and therapy [69–72].

Bile acids (BAs), which are steroidal compounds metabolized by gut microbiota, also play a significant role in CRC. Secondary BAs have been shown to compromise the integrity of the colonic barrier, induce oxidative DNA damage, promote inflammatory responses, and activate the NF-κB signaling pathway, all of which contribute to CRC progression. Specific bacterial genera, including *Clostridium* and *Eubacterium*, are involved in BA transformations, such as 7α-dehydroxylation and sulfidation, which further influence the gut microenvironment and CRC development [73]. Additionally, cholecystectomy may be associated with CRC through microbiota imbalances and altered BA production, although the mechanisms require further investigation [74, 75].

The gut microbiota also produces a wide range of other metabolites that influence host physiology, immunity, and cancer development. Among these metabolites, β-galactosidase (β-gal), tryptophan catabolites, and trimethylamine N-oxide (TMAO) have attracted attention due to their role in the pathogenesis, progression, and potential as biomarkers or therapeutic targets of CRC. β-D-Galactosidases, commonly referred to as lactases, are enzymes that hydrolyze the β (1 ⟶ 4) glycosidic bond in lactose, yielding glucose and galactose. In addition to this hydrolytic activity, they possess transgalactosylation capacity, enabling the synthesis of galacto-oligosaccharides (GOS) through the transfer of galactose residues to other lactose molecules. GOS are recognized as functional prebiotic compounds that selectively stimulate the growth of Bifidobacteria in the human intestine. The predominance of Bifidobacteria contributes to several health-promoting effects, including suppression of harmful bacteria, regulation of bowel movements, reduction of intestinal ammonia, enhancement of mineral absorption, modulation of cholesterol and lipid levels, and potential protection against CRC. Due to these beneficial properties, GOS are widely utilized as low-calorie sweeteners and bioactive ingredients in various food products, including fermented dairy items, confectionery, baked goods, and beverages [76–78].

Similarly, tryptophan (Trp) metabolism plays a dual role in cancer. Trp is metabolized via the kynurenine, indole, and serotonin pathways. In CRC, indole-3-acetaldehyde (IAAD) exhibits dose-dependent effects: Low concentrations inhibit tumor growth, while high levels enhance invasion through AhR activation and interaction with metastasis-associated proteins. Kynurenine, a key immunosuppressive metabolite, drives T-cell dysfunction and tumor progression. Interventions targeting Trp metabolism—such as IDO/TDO enzyme inhibitors or microbiome-based modulation—have shown promise, particularly in enhancing immunotherapy. However, tumor heterogeneity and complex host-microbe interactions pose challenges in disease transmission [79–82].

TMAO, which is derived from the conversion of choline and carnitine by gut microbes, also links diet, microbiota, and disease. Its elevated levels are associated with systemic inflammation, metabolic dysfunction, and several chronic diseases, including CRC. Mechanistically, TMAO activates proinflammatory pathways such as NF-κB and the NLRP3 inflammasome while disrupting epithelial barriers through the HULC/p38 MAPK axis. Although some studies have shown a correlation between TMAO and CRC progression, the results are inconsistent across populations. Interestingly, TMAO may also enhance antitumor immunity in some contexts, suggesting that its effects are context- and concentration-dependent. Monitoring TMAO via LC–MS or novel electrochemical biosensors has diagnostic potential [83–86]. β-Gal, Trp metabolites, and TMAO together demonstrate the multifaceted roles of gut microbiota-derived factors in CRC. Each of them represents a unique intersection of microbial metabolism, immune regulation, and cancer biology, providing not only insight into tumorigenesis but also potential targets for diagnosis, prognosis, and therapeutic innovations.

## 4. Gut Microbiota Biomarkers for Early, Noninvasive CRC Detection

The majority of CRC cases are diagnosed at intermediate or advanced stages, frequently requiring surgical intervention, which is associated with increased mortality risks. Colonoscopy is regarded as the gold standard for CRC screening and has been shown to significantly reduce mortality rates. However, its invasive nature poses a barrier to widespread implementation and patient compliance [53, 87, 88]. Noninvasive alternatives, such as gFOBTs, FITs, and blood DNA methylation biomarkers, are more patient-friendly and cost-effective but less sensitive in detecting advanced CRC and CRA [89]. Emerging research highlights the gut microbiome's pivotal role in CRC development and progression. Stool and mucus sample analyses reveal microbiome changes associated with different stages of CRC [90–92]. The considerable variability in gut microbiota composition, influenced by factors such as medication use, genetic predisposition, age, diet, and lifestyle, presents a challenge in identifying specific microbial species predictive of CRC. Despite these complexities, advancements have been made in this field. A 2014 study examining stool microbiomes from CRC patients, individuals with colon adenoma, and healthy controls integrated microbiome data with clinical risk factors—such as age, race, and body mass index (BMI)—to develop a predictive classification algorithm. This integrative approach enhanced the accuracy of CRC prediction beyond models based solely on clinical factors. However, the study relied on 16S rRNA gene sequencing and lacked independent validation, highlighting the need for further research to confirm its findings [93].

### 4.1. Bacterial, Viral, and Fungal Biomarkers

Recent research has highlighted the potential of microbiome-associated biomarkers for noninvasive CRC detection, particularly in early stages [94, 95]. Key candidates include *E. coli*, *F. nucleatum*, and *B. fragilis*, which have shown promise in early detection, risk assessment, and treatment prediction [96, 97]. Elevated *F. nucleatum* levels are notably linked to CRC and precancerous lesions, with increased abundance in tumor tissues and adjacent areas. Alterations in the relative abundance of *F. nucleatum* in comparison to *F. prausnitzii* and *Bifidobacterium* in fecal samples may serve as potential early biomarkers for the detection of CRC [98].

A study conducted in China demonstrated that the detection of *F. nucleatum* DNA in saliva exhibited greater diagnostic accuracy for CRC compared to conventional tumor markers, such as CEA and CA19-9. Moreover, the levels of *F. nucleatum* DNA were found to be associated with patient survival outcomes, suggesting its potential as a prognostic biomarker [99]. Additional research identified *Parvimonas micra* and *Solobacterium moorei* as CRC-associated species and validated 20 microbial gene markers across cohorts in France and Austria. Two markers—*F. nucleatum*'s butyryl-CoA dehydrogenase and *P. micra*'s RNA polymerase β subunit—distinguished CRC microbiomes with high precision (AUC: 0.84) [5, 94]. Combining microbial markers with FITs notably increases the diagnostic sensitivity of CRC from 73.1% to 92.3%. Adding species like *Bacteroides clarus*, *Roseburia intestinalis*, and *Clostridium hathewayi* further enhances diagnostic accuracy [100]. While microbial markers hold promise, challenges persist due to variability across populations and an emphasis on advanced stages of CRC.

Early-stage CRC research has also revealed changes in microbiota. A 2019 Japanese study of 631 participants identified *F. nucleatum*, *Atopobium parvulum*, and *Actinomyces odontolyticus* as enriched in polyps and stage 0 CRC, alongside metabolites like branched-chain amino acids and deoxycholic acid (DCA), which effectively distinguished stage 0 CRC from controls [101]. The gut virome and mycobiome also offer potential biomarkers for CRC. A study found 22 viral taxa discriminating CRC cases (AUC: 0.8), while fungal biomarkers like *Malassezia* enrichment and reduced *Saccharomycetes* showed strong diagnostic potential (AUC: 0.93) [40, 43, 102]. Combining bacterial and fungal markers, such as *A. rambelli* with *F. nucleatum*, further improves diagnostic accuracy by 1.4%–10.6%.

Microbial biomarkers, when integrated with conventional clinical methods, offer significant potential to enhance CRC screening and early detection, although further validation and refinement are needed.

## 5. The Intestinal Microbiome Influences CRC Prevention and Therapy

Considering the significant role of gut microbiota in CRC, research has increasingly focused on microbiome modulation as a strategy for reducing CRC risk. Diet and lifestyle factors play a pivotal role in shaping gut microbial composition and metabolic activity, thereby influencing CRC development. Notably, inadequate nutrition, particularly a high-fat diet, has been associated with an increased risk of colorectal tumor formation. This correlation is primarily attributed to gut dysbiosis, characterized by an overgrowth of pathogenic bacteria and the production of harmful metabolites, such as lysophosphatidic acid, which contribute to tumorigenesis [103]. In contrast, a fiber-rich diet promotes the production of SCFAs by beneficial bacteria such as *E. rectale* and *Clostridium symbiosum*. SCFAs help modulate immunity and reduce inflammation, protecting against CRC [104]. A meta-analysis revealed that regular physical activity is associated with a reduced risk of CRC, potentially through its role in enhancing gut microbiome diversity and promoting the growth of SCFA-producing bacteria [105–108].

Nevertheless, uncertainties persist concerning the optimal type, intensity, and duration of exercise for CRC prevention, underscoring the necessity for further preclinical and clinical investigations. Additionally, the gut microbiome plays a crucial role in modulating the efficacy and toxicity of anticancer and immunotherapy treatments through mechanisms such as drug metabolism and immune regulation, highlighting its significance in influencing cancer treatment outcomes.

### 5.1. The Intestinal Microbiota and Chemotherapy Response

The intestinal microbiota has a significant impact on chemotherapy efficacy, drug resistance, and gastrointestinal toxicity. It influences the effectiveness of chemotherapy agents like 5-fluorouracil (5-FU), gemcitabine, and platinum compounds through mechanisms such as immune modulation, metabolic activity, and changes in microbial diversity [109, 110]. CRC patients who respond well to chemotherapy tend to have higher levels of *Sutterella* and *Roseburia* in their microbiota. In contrast, increased *Fusobacterium* levels are linked to poor responses and worse outcomes [111]. The microbiome also contributes to chemoresistance, with *F. nucleatum* being more prevalent in CRC tissues from patients with recurrence after chemotherapy. High *F. nucleatum* levels are associated with resistance to platinum-based agents and 5-FU by modulating cancer cell autophagy [16, 61, 112]. Targeting *F. nucleatum* before chemotherapy may enhance treatment effectiveness, making it a key marker for predicting chemotherapy response and CRC outcomes [113].

Chemotherapy can alter the gut microbiome, affecting drug metabolism. Studies show that antibiotic-treated mice experience reduced effectiveness of platinum chemotherapy, as their gut microbiota is crucial for platinum-induced DNA damage and cancer cell death [114]. Antibiotics reduce proinflammatory gene expression and impair ROS production, which are essential for the tumor-killing effects of platinum drugs. The microbiome also impacts irinotecan (CPT-11) metabolism. *Bacterial β-glucuronidase* reactivates irinotecan's active compound (SN-38) in the gut, contributing to intestinal damage and severe diarrhea. Inhibiting this enzyme could reduce irinotecan's toxicity and improve treatment outcomes [115–118]. These findings highlight the potential to manipulate the microbiome to enhance chemotherapy efficacy and reduce side effects.

### 5.2. The Gut Microbiome and Immunotherapy Response

Immunotherapy enhances the immune system's ability to identify and target cancer cells more effectively. It has been tailored to specific cancer types and stages to overcome mechanisms that allow tumors to evade immune detection. While not universally effective, it has significantly improved treatment outcomes for particular individuals and cancer types [119]. The intestinal microbiome plays a crucial role in immune responses and influences the success of immune checkpoint inhibitors (ICIs) targeting pathways like PD-1–PD-L1 and CTLA-4 [120–123]. A study investigating the role of commensal bacteria in tumor-associated inflammatory processes found that disruption of the microbiota, either through ABX or in GF mice, impaired tumor responses to CpG-oligonucleotide immunotherapy and platinum-based chemotherapy, including oxaliplatin and cisplatin. In both ABX-treated and GF mice, myeloid-derived cells infiltrating tumors exhibited reduced cytokine production following CpG-oligonucleotide treatment, along with diminished ROS generation and cytotoxicity after chemotherapy. Gene expression analysis of pretherapy tumors in ABX-treated mice showed a downregulation of genes associated with inflammation, phagocytosis, antigen presentation, and adaptive immunity, while genes linked to tissue development, cancer progression, and metabolism were upregulated. The diminished effectiveness of anti-IL-10R/CpG-ODN immunotherapy in ABX-treated mice was found to be independent of adaptive immunity but reliant on tumor necrosis factor (TNF), which was similarly reduced in GF mice. Administration of LPS to ABX-treated mice restored TNF production, suggesting that bacterial components play a role in priming myeloid cells for TLR9-dependent responses. Fecal microbiota analysis identified specific bacterial genera associated with these immune responses, with *Alistipes* positively correlated with TNF production and *Lactobacillus* negatively correlated. Oral supplementation with *Alistipes shahii* restored TNF production, whereas *Lactobacillus fermentum* suppressed immune responses in intact mice. Moreover, both ABX and GF conditions reduced the efficacy of oxaliplatin and cisplatin, likely due to impaired early cytotoxicity and decreased monocyte activation. These findings indicate that the gut microbiota significantly influence chemotherapy-induced ROS production, particularly through tumor-associated inflammatory cells, with MyD88 playing a role in the early antitumor effects of oxaliplatin. Notably, tumors from ABX-treated mice exhibited reduced oxaliplatin-induced DNA damage despite similar platinum binding, suggesting a microbiota-mediated mechanism regulating ROS-dependent DNA damage. This study underscores the critical role of the microbiome in modulating both immunotherapy and chemotherapy efficacy [114].

A commentary on the study by Routy et al. examined the influence of gut microbiome composition on responses to PD-1-based immunotherapy in epithelial tumors. The research revealed that primary resistance to ICIs was associated with gut dysbiosis. Notably, FMT, or oral administration of *Akkermansia muciniphila,* restored sensitivity to anti-PD-1 therapy in mouse models. This restoration was mediated by interleukin-12 (IL-12) and increased recruitment of CCR9^+^CXCR3^+^CD4^+^ T lymphocytes. A proposed mechanistic model suggests that *A. muciniphila* exerts dual effects on both cancer and immune cells by producing SCFAs, such as acetate and propionate, which possess tumor-suppressive properties. SCFAs promote histone hyperacetylation, activate p21, downregulate inhibitor of apoptosis (IAP) proteins, and enhance caspase activity, ultimately inducing apoptosis. Additionally, SCFAs modulate immune responses by activating the mTOR-S6K and STAT3 signaling pathways in T cells, thereby promoting the differentiation of Th17, Th1, and IL-10^+^ regulatory T cells. These findings highlight the potential of microbiota-based interventions to enhance ICI efficacy and suggest novel biomarkers for predicting treatment response [120].

Another investigation into the microbiota's influence on anti-PD-L1 immunotherapy found that distinct microbiota compositions in mice from different facilities (JAX and TAC) influenced melanoma growth and immune responses. JAX mice exhibited superior antitumor immunity, which was transferable through cohousing or fecal transfer. Administration of JAX feces to TAC mice with established tumors improved tumor control and increased CD8+ T cell infiltration, with effects comparable to anti-PD-L1 therapy. Combination treatment nearly abolished tumor progression. Sequencing identified *Bifidobacterium* as a key immunomodulatory microbe, with oral supplementation of *Bifidobacterium* species enhancing tumor-specific CD8+ T cell responses. The effects depended on live bacteria and were abrogated upon depletion of CD8+ T cells. Transcriptional profiling of tumor-infiltrating dendritic cells (DCs) from *Bifidobacterium*-treated mice revealed upregulation of genes involved in cytokine signaling, T cell activation, and DC maturation. The study concluded that commensal *Bifidobacterium* enhances DC activation, leading to improved CD8+ T cell effector function and greater antitumor efficacy of anti-PD-L1 therapy [121].

Further research on CTLA-4 blockade immunotherapy revealed that its antitumor effects were contingent on gut microbiota. While CTLA-4 blockade induced tumor regression in specific pathogen-free (SPF) mice, it failed in GF or antibiotic-treated mice, demonstrating the microbiota's necessity for immune activation. The therapy altered the fecal microbiome, depleting *Bacteroidales* and *Burkholderiales* while enriching *Clostridiales*. Recolonization of GF or antibiotic-treated mice with *B. fragilis* and *Burkholderia cepacia* restored therapeutic efficacy. *B. fragilis*-mediated TH1 responses were crucial for CTLA-4 blockade, as adoptive transfer of *B. fragilis*-specific TH1 cells reinstated immunotherapy effectiveness in GF mice. In melanoma patients, responders to CTLA-4 blockade exhibited enriched *Bacteroides* spp., with fecal transplantation from these individuals improving response in GF mice. These findings suggest that microbiota composition influences IL-12-dependent TH1 immune responses, which are critical for effective CTLA-4 blockade [123].

A study investigating the impact of intestinal microbiota on relapse risk following allogeneic hematopoietic cell transplantation (allo-HCT) analyzed data from 541 patients. The findings demonstrated that a bacterial group dominated by *Eubacterium limosum* was associated with a reduced risk of disease relapse or progression (POD). This association remained significant in multivariable analyses and was particularly evident in recipients of T-cell–replete allografts. Furthermore, a higher abundance of *E. limosum* correlated with improved overall survival; however, no significant association was observed with acute graft-versus-host disease (GVHD) or transplant-related mortality. The study suggested that distinct microbiota compositions may influence graft-versus-tumor (GVT) activity, highlighting their potential as biomarkers for assessing relapse risk in allo-HCT recipients [124].

A review of three studies (Routy et al., Gopalakrishnan et al., and Matson et al.) assessing gut microbiota's role in PD-1 immunotherapy response revealed inconsistencies in the identified bacterial taxa associated with treatment efficacy. Routy et al. [120] identified *Akkermansia*, Gopalakrishnan et al. [122] reported *Faecalibacterium*, and Matson et al. [125] found *Bifidobacterium* as predictive of response. To address discrepancies, a re-analysis using a common computational pipeline (QIIME and MetaPhlAn2) was conducted, revealing that while individual datasets reproduced some original findings, no consistent bacterial taxa were identified across studies. However, metagenomic functional analysis suggested that microbial gene content and function might have better predictive value than community composition alone. The review emphasized the need for standardized microbiota sampling and larger clinical trials to elucidate the functional mechanisms underlying microbiota-mediated immunotherapy responses.

A 2019 study identified 11 bacterial strains that enhanced the effectiveness of ICIs in mice, emphasizing the need for consistent microbial signatures to predict patient responses to immunotherapy [126]. While immunotherapy is not universally effective for all CRC subtypes, it shows promise for patients with microsatellite instability-high (MSI-H) or DNA mismatch repair-deficient (dMMR) CRC, which are highly responsive to PD-1 blockade [127–129]. Ongoing research seeks to harness the gut microbiota to improve immunotherapy effectiveness and minimize side effects, particularly in CRC patients [130, 131].

### 5.3. The Gut Microbiome Plays a Critical Role in Cancer Prevention

Recent studies have revealed a significant gap in the current literature regarding the specific interactions between gut microbiota and targeted therapeutic agents in CRC. While the role of gut microbiota has been extensively investigated in chemotherapy, immunotherapy, and radiotherapy, its impact on the efficacy, resistance, and safety of molecularly targeted therapies remains underexplored. This review examines the influence of specific microbial taxa and their metabolic products on key oncogenic signaling pathways, emphasizing their capacity to mediate both tumor-suppressive and tumor-promoting effects. Mounting evidence indicates that particular bacterial species and their metabolites can modulate the effectiveness of targeted therapies, particularly those that inhibit the epidermal growth factor receptor (EGFR), vascular endothelial growth factor (VEGF), and Kirsten rat sarcoma viral oncogene homolog (KRAS) signaling cascades [132]. Several microbial-derived compounds exhibit notable antitumor activities. Ursodeoxycholic acid (UDCA), predominantly produced by *Parabacteroides distasonis*, has been shown to suppress EGFR expression and inhibit the PI3K/AKT pathway, thereby triggering apoptosis and attenuating EMT in malignant cells. Butyrate, a major SCFA synthesized by commensal gut bacteria, reduces VEGF and neuropilin-1 (NRP-1) expression, impairs angiogenesis, and elevates ROS production. This oxidative shift subsequently suppresses the PI3K/AKT/mTOR signaling axis, promoting autophagy in tumor cells. Similarly, propionate contributes to antitumor activity by inhibiting the mTOR pathway, also resulting in autophagic responses [132–134].

In contrast, other microbial metabolites have been implicated in the progression and aggressiveness of CRC. DCA, a secondary BA produced by *Clostridium sordellii*, enhances EGFR activation and stimulates PI3K/AKT/mTOR signaling, leading to increased tumor cell proliferation, invasion, and angiogenesis through upregulation of VEGF mRNA. TMAO has also been found to amplify VEGF expression and secretion, thereby facilitating tumor neovascularization. *F. nucleatum*, frequently enriched in CRC tissue, contributes to EMT through activation of the EGFR pathway. Notably, this bacterium has been associated with resistance to cetuximab—an anti-EGFR monoclonal antibody—by activating the PI3K/AKT and JAK/STAT3 signaling pathways, resulting in elevated half-maximal inhibitory concentrations (IC50) in infected tumor cells [135]. Additionally, LPS derived from Gram-negative bacteria upregulates VEGFR expression and fosters cellular migration and invasion while simultaneously enhancing PI3K/AKT/mTOR signaling and suppressing autophagic activity [132, 136].

Beyond influencing therapeutic efficacy, the gut microbiota also plays a central role in the emergence of drug resistance to targeted therapies. KRAS mutations, present in approximately 45% of CRC patients, are strongly associated with reduced responsiveness to EGFR-targeted agents, such as cetuximab [137]. These mutations result in the constitutive activation of downstream effectors, including Raf kinase and PI3K, regardless of upstream receptor inhibition [138]. The EGFR pathway itself is also recognized as a contributor to resistance mechanisms against KRAS G12C inhibitors, suggesting that combination strategies targeting both EGFR and mutant KRAS may be necessary to overcome resistance [137]. In this context, the gut microbiota may indirectly enhance the efficacy of anti-KRAS therapies by modulating systemic metabolic pathways. For example, dietary fiber-induced increases in *Prevotella* abundance have been linked to improved glucose metabolism, which may mitigate resistance to KRAS-targeted agents [132]. Moreover, therapeutic resistance, which occurs in nearly 80% of advanced CRC cases, is frequently mediated through the compensatory activation of alternative pathways, such as the PI3K/Akt and Ras/Raf/MEK/ERK pathways, both of which are closely interconnected with EGFR signaling. Additionally, hypoxic conditions, driven by the stabilization of hypoxia-inducible factor 1-alpha (HIF-1α), have been shown to enhance the expression of KRAS G12V, further contributing to resistance against anti-EGFR treatments [139].

The gut microbiota is also increasingly recognized as a determinant of the safety and tolerability of targeted cancer therapies. A notable example is tyrosine kinase inhibitor (TKI)-induced diarrhea, a common adverse event that may significantly impair treatment adherence. FMT has demonstrated beneficial effects in alleviating TKI-related gastrointestinal toxicity, particularly in patients with metastatic renal cell carcinoma. Likewise, targeted ABX against Gram-negative organisms has proven effective in preventing neratinib-induced diarrhea. Microbial profiling of affected patients has revealed increased levels of *Bacteroides* species and a concurrent decrease in *Prevotella* and butyrate-producing bacteria. Elevated abundances of *Bacteroidetes* and *Firmicutes* have also been detected in individuals experiencing severe adverse reactions to EGFR-TKIs, suggesting that these microbial signatures could serve as predictive markers for toxicity. These findings underscore the therapeutic potential of microbiota modulation strategies, such as FMT, probiotics, or bacteriophage-based interventions, for improving the safety profile of targeted therapies [132].

In addition to these well-characterized mechanisms, emerging studies have begun to investigate the role of less common microbial species in modulating treatment outcomes. For instance, *Klebsiella quasipneumoniae* and *Veillonella* species have been identified as potential contributors to therapeutic response variability in metastatic CRC, although further research is required to validate their clinical significance [140]. Furthermore, both probiotics and SCFAs are generally considered safe and potentially beneficial adjuncts to conventional CRC treatment regimens [136].

## 6. Gut Microbiome Modulation for CRC Prevention and Management

**Figure 3:**
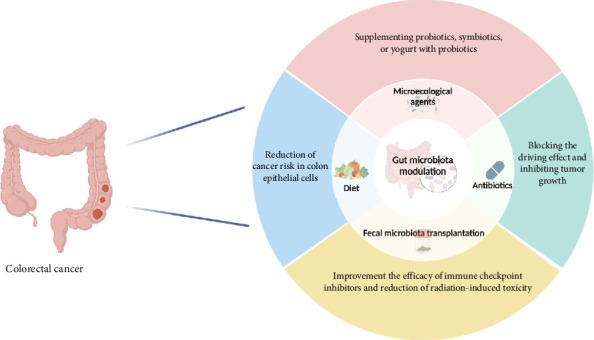
An exhaustive investigation of potential methods to manipulate the intestinal microbiome for CRC prevention and treatment, including dietary alterations, microbial component alterations, fecal microbiota transplantation (FMT), and antibiotic interventions.

Preventive approaches, including dietary modifications, probiotic supplementation, and antibiotic administration, have demonstrated potential in lowering the risk of CRC. Epidemiological research has identified modifiable risk factors, such as dietary habits, obesity, and lifestyle choices, which contribute to CRC development. Furthermore, growing evidence suggests that microecological interventions, such as FMT, can enhance therapeutic efficacy and clinical outcomes by influencing gut microbiota composition. These microbiome-based strategies are anticipated to play a crucial role in the prevention and management of CRC [22, 141] (Figure 3).

### 6.1. Nutritional Modifications

Diet significantly influences the gut microbiota, with variations in dietary patterns across populations linked to different CRC risks. For example, a study comparing African Americans and rural Africans found that higher levels of *Bacteroides* in African Americans were associated with greater animal protein and fat intake, and lower fiber consumption [142, 143]. Dietary changes, such as adopting a high-fiber, low-fat diet, can alter gut microbiota composition in African Americans, reducing harmful bacteria, increasing beneficial populations, boosting butyrate production and lowering secondary BA levels. These changes reduce the risk of colon epithelial cell carcinogenesis [144]. Studies show that even short-term dietary modifications, like a 2-week high-fiber, low-fat diet, can reduce inflammation and cancer-related biomarkers in African Americans. Conversely, switching rural Africans to a high-fat, low-fiber diet leads to opposite effects, increasing cancer risk markers [104].

Dietary components influence CRC onset and progression through their impact on gut microbiota and biochemical production. Approaches like nutraceutical supplements containing probiotics or bioactive compounds can help maintain intestinal integrity and support a balanced microbiome-host interaction [145].

## 7. Probiotics

Probiotics are beneficial live microorganisms that provide health advantages when taken in sufficient quantities. They have been studied for their immunemodulating and anticancer properties, particularly in CRC prevention [146, 147]. Probiotics promote the growth of beneficial bacteria, enhance SCFA production, reduce harmful microorganisms, decrease inflammation, and encourage apoptosis, all of which help prevent CRC progression.

Preclinical studies show that probiotic strains like *Bifidobacterium* and *Lactobacillus* inhibit cancer cell growth, induce cell death, regulate immune responses, detoxify carcinogens, and produce compounds that counteract carcinogenesis [148–150]. Specific strains also reduce the activity of enzymes that convert carcinogens into potent cancer-causing agents. The outer layers of probiotic bacteria, including peptidoglycan, polysaccharides, and glycoproteins, may help detoxify harmful substances and prevent colon cancer [151, 152]. Probiotics also regulate the immune system and inhibit CRC progression. For instance, *Clostridium butyricum* treatment in mice reduced tumor incidence, decreased Th2 and Th17 cells, and suppressed T lymphocyte activity. They can interfere with cell division, reduce inflammatory molecules like NF-κB and IL-22, and promote tumor cell death [153]. By producing anti-inflammatory molecules, antioxidant compounds, and SCFAs, probiotics help maintain the intestinal barrier's integrity [154, 155]. While probiotics primarily serve a preventive role in healthy individuals, they can also regulate the gut microbiome in CRC patients, potentially enhancing the effectiveness of CRC treatments alongside other therapies.

## 8. Antibiotic

Antibiotics can significantly alter the gut microbiome, which may influence CRC development. Studies in rat models have shown that antibiotics can decrease crypt height and increase CRC risk, particularly when combined with heme intake [156]. Antibiotics like penicillins and quinolones, known for their antianaerobic or antiaerobic properties, are associated with a higher CRC risk, which increases with higher doses [157]. A U.K. study found that repeated penicillin treatments, promoting bacterial or fungal overgrowth, slightly raised CRC risk [158].

Additionally, research indicates that altering the microbiome with antibiotics during early inflammation can reduce tumor formation in rodent models. In a mouse model of CRC induced by AOM and DSS, microbiome changes caused by antibiotics led to fewer colon tumors, but this effect was only seen when the microbiome changes persisted throughout the inflammation phase [159]. However, antibiotic use in early childhood has been linked to a higher risk of colonic adenomas, which can later develop into CRC, suggesting long-term gut microbiome imbalances may contribute to CRC risk [52, 160]. Antibiotics may also reduce the tumor-promoting effects of bacteria like *F. nucleatum* and *E. coli*, which are associated with CRC. For instance, metronidazole treatment has been shown to lower *F. nucleatum* levels in early CRC lesions and liver metastases, slowing tumor progression, indicating potential therapeutic benefits for CRC linked to *F. nucleatum* infections [14]. Further research is needed to understand the impact of antibiotics on the microbiome and their role in CRC development, which could inform targeted therapies and improve CRC understanding.

## 9. Conclusion

The global incidence and mortality rates of CRC have been increasing in recent years, with disruptions in the intestinal microbiome playing a significant role in its progression. Specific microbial species, such as *F. nucleatum*, *pks + E. coli*, and ETBF, have been implicated in CRC development. However, the potential contributions of fungal and viral communities remain poorly understood and warrant further investigation. The microbial factors associated with CRC are linked to gut microbiome dysbiosis, pathogen colonization, microbial metabolites, and virulence factors, which activate inflammatory pathways and induce DNA damage in intestinal cells, thereby promoting CRC progression. Nevertheless, further research, including laboratory experiments and animal model studies, is essential to elucidate the precise role of the microbiome in CRC pathogenesis. An emerging area of interest is the application of microbial biomarkers for the early detection and prediction of CRC. Integrating these biomarkers with conventional clinical screening methods may significantly improve CRC diagnosis and prevention strategies.

Furthermore, interventions targeting the gut microbiota, such as modifying microecological factors, may improve cancer treatment effectiveness, reduce drug resistance, and mitigate the severe gastrointestinal side effects of CRC therapies. Ongoing research and international collaboration are crucial to identifying carcinogenic pathways, discovering new biomarkers, and developing effective microbiome-based interventions for CRC.

## Nomenclature

SCFAs: Short-chain fatty acids
ROS: Reactive oxygen species
MSI-H: Microsatellite instability-high
dMMR: DNA mismatch repair-deficient
5-FU: 5-Fluorouracil
TH1: T helper cells
FIT: Fecal immunochemical test
gFOBTs: Fecal occult blood tests
AOM: Azoxymethane
DSS: Dextran sodium sulfate
Bas: Bile acids
IBD: Inflammatory bowel disease
pks: Polyketide synthase
FMT: Fecal microbiota transplantation
